# Biotin and chromium histidinate improve glucose metabolism and proteins expression levels of IRS-1, PPAR-γ, and NF-κB in exercise-trained rats

**DOI:** 10.1186/s12970-018-0249-4

**Published:** 2018-09-15

**Authors:** Mine Turgut, Vedat Cinar, Ragip Pala, Mehmet Tuzcu, Cemal Orhan, Hafize Telceken, Nurhan Sahin, Patrick Brice Defo Deeh, James R. Komorowski, Kazim Sahin

**Affiliations:** 1 Faculty of Sports Sciences, Firat University, Elazig, Turkey; 2 Department of Biology, Faculty of Science, Firat University, 23119 Elazig, Turkey; 3 Department of Animal Nutrition, Faculty of Veterinary Medicine, Firat University, Elazig, Turkey; 4 Animal Physiology and Phytopharmacology Laboratory, University of Dschang, Dschang, Cameroon; 5 Scientific and Regulatory Affairs, Nutrition 21 Inc, 1 Manhattanville Road, Purchase, NY 10577 USA

**Keywords:** Chromium Histidinate, Biotin, PPAR-γ, IRS-1, NF-κB

## Abstract

**Background:**

Chromium histidinate (CrHis) and biotin are micronutrients commonly used to improve health by athletes and control glycaemia by patients with diabetes. This study investigates the effects of 8-week regular exercise training in rats together with dietary CrHis and biotin supplementation on glucose, lipids and transaminases levels, as well as protein expression levels of peroxisome proliferator-activated receptor gamma (PPAR-γ), insulin receptor substrate-1 (IRS-1) and nuclear transcription factor kappa B (NF-κB).

**Methods:**

A total of 56 male Wistar rats were randomly divided into 8 groups of 7 animals each and treated as follows: Control, CrHis, Biotin, CrHis+Biotin, Exercise, CrHis+Exercise, Biotin+Exercise, and CrHis+Biotin+Exercise. The doses of CrHis and biotin were 400 μg/kg and 6 mg/kg of diet, respectively. The training program consisted of running at 30 m/min for 30 min/day at 0% grade level, 5 days per week, once a day for 6 weeks. Serum glucose, total cholesterol (TC), high-density lipoprotein cholesterol (HDL), triglycerides (TG), aspartate aminotransferase (AST) and alanine aminotransferase (ALT) levels were measured with an automatic biochemical analyzer. Muscle and liver PPAR-γ, IRS-1 and NF-κB expressions were detected with real-time polymerase chain reaction.

**Results:**

Regular exercise significantly (*p* < 0.001) decreased glucose, TC and TG levels, but increased HDL cholesterol. Dietary CrHis and biotin supplementation exhibited a significant (*p* < 0.001) decrease in glucose (effect size = large; ƞ2 = 0.773) and TG (effect size = large; ƞ2 = 0.802) levels, and increase in HDL cholesterol compared with the exercise group. No significant change in AST and ALT (effect size = none) levels was recorded in all groups (*p* > 0.05). CrHis/biotin improves the proteins expression levels of IRS-1, PPAR-γ, and NF-κB (effect size: large for all) in the liver and muscle of sedentary and regular exercise-trained rats (*p* < 0.001).

**Conclusions:**

CrHis/biotin supplementation improved serum glucose and lipid levels as well as proteins expression levels of PPAR-γ, IRS-1 and NF-κB in the liver and muscle of exercise-trained rats, with the highest efficiency when administered together. CrHis/biotin may represent an effective nutritional therapy to improve health.

## Background

Exercise is an important part of a healthy lifestyle which decreases the risk of various metabolic diseases over the long term [1, 2]. Indeed, exercise is known to have preventive as well as curative effects for metabolic diseases due to its capacity for burning fat in our body [3, 4]. For instance, it has been reported that exercise controls insulin and glucose homeostasis, increases fatty acid oxidation in muscles [5–7] and decreases blood glucose level [8]. Exercise has also been shown to reduce systemic inflammation [9], boost immune cell function [10, 11] and prevent type 2 diabetes by approximately 50%, either as a lone treatment [12] or in combination with other therapies [13, 14]. In addition, in healthy patients, exercise such as weightlifting or distance running can elevate aspartate aminotransferase (AST) and alanine aminotransferase (ALT) levels, indicators of liver and muscle inflammation [15]. Moreover, exercise decreases triglycerides and increases HDL levels [16], and modulates various biomarkers such as peroxisome proliferator-activated receptors (PPAR-γ), insulin receptor substrate 1 (IRS-1) and nuclear transcription factor kappa B (NF-κB) in the body [17, 18]. PPAR-γ, IRS-1 and NF-κB are important biomarkers involved in mediating numerous physiological process including insulin signalling, glucose and lipid metabolism, and inflammation [19, 20]. Exercise stimulates PPAR-γ signalling events and upregulates genes related to lipid metabolism [21], enhances insulin activation of IRS*-*1-associated PI3-kinase in human skeletal muscle [22] and improves intramuscular NF-κB signalling [23]. The combination of exercise and micronutrients such as chromium and biotin are commonly used to improve performance by athletes [24] and ameliorate glycemic control by patients with diabetes [25, 26].

Chromium histidinate (CrHis) is an organic complex of chromium mineral formed by histidine amino acid. CrHis is a safe and highly absorptive compound [26, 27], commonly used to improve muscle mass and protect against various metabolic diseases such as obesity [28] and diabetes mellitus [29]. It has been shown that chromium deficiencies contribute to hyperglycemia, insulin resistance and dyslipidemia, mainly in patients with type 2 diabetes [30, 31]. Biotin, a water-soluble vitamin synthesized by plants and microorganisms, is an important vitamin [32]. It modulates glucose-stimulated insulin secretion, glucose uptake in the liver and glycogen synthesis in the presence of high plasma glucose level [32]. Chromium picolinate (CrPic) and the combination of CrPic and biotin were shown to enhance glucose disposal, decrease total cholesterol and increase HDL-cholesterol concentrations in type 2 diabetes patients [25, 33, 34]. In skeletal muscle culture, CrPic and biotin have been shown to greatly stimulate glucose uptake and glycogen production when these nutrients were combined [35].

To the best of our knowledge, no experimental works on the effects of exercise combined with CrHis and biotin supplementation have been published. Therefore, the present study was undertaken to investigate the effects of dietary supplementation with CrHis (400 μg/kg/day), biotin (6 mg/kg/day) and their combination on serum glucose and lipid levels as well as liver transaminases (ALT and AST) levels in sedentary and exercise-trained rats. The proteins expression levels of PPAR-γ, IRS-1 and NF-κB were also evaluated.

## Methods

### Animals

Male Wistar rats (8 weeks, 200–220 g body weight) were obtained from the Firat University Experimental Research Center (FÜDAM) and used in this study. Rats were maintained in a standard laboratory environment (temperature: 22 ± 2 °C, relative humidity: 55 ± 5% and 12/12 h light/dark cycle). Animals were given a standard diet which was refreshed daily on a regular basis and their hygiene was maintained. The experiment was carried out at FUDAM. This study was approved by the Animal Ethical Committee of Firat University (FÜHADEK) (2014/05/50). All experiments were performed in accordance with the internationally accepted standard ethical guidelines for laboratory animal use and care as described in the European community guidelines; EEC Directive 86/609/EEC, of the 24th November 1986 [36].

### Experimental design

A total of 56 male Wistar rats were divided into 8 groups of 7 animals each and treated as follows: Group 1 (Control), rats fed a standard diet and no exercised; Group 2 (CrHis), rats fed a standard diet containing CrHis and no exercised; Group 3 (Biotin), rats fed a standard diet containing biotin and no exercised; Group 4 (CrHis+Bio), rats fed a standard diet containing CrHis/biotin and no exercised; Group 5 (Exercise), rats fed a standard diet and exercised; Group 6 (E + CrHis), rats fed a standard diet containing CrHis and exercised; Group 7 (E + Bio), rats fed a standard diet containing biotin and exercised; Group 8 (E + CrHis+Bio), rats fed a standard diet containing CrHis/biotin and exercised. In groups, 5–8 animals were exercised 5 days a week for 6 weeks. In all groups, the rats were allowed free access to standard diet and drinking water for 6 weeks. The standard diet was composed, according to the American Institute of Nutrition (AIN)-93 [37], recommendations, of casein (20%), soyabean oil (7%), wheat starch (53·2%), sucrose (10%), potato starch (5%), L-cysteine (0·3%), vitamin mix AIN-93 M (1%) and mineral mix AIN-93 M (3·5%). The analytically determined Cr content in standard and Cr-supplemented diet was 1.12 and 1.48 mg/kg of diet. In addition, biotin levels in the diets were 0.26 and 6.15 mg/kg of diet. The intake of Cr, and biotin based on an analytical assessment of the diets are shown in Table 2. The doses of CrHis (400 μg/kg) and biotin (6 mg/kg) were based on our previous study [38, 39].

The exercise was performed on a motor-driven treadmill (Treadmill, MAY- TME 0804, Commat Limited, Ankara). The exercising animals were familiarized with the treadmill and ran at 10 m/min for 3 days until the initiation of the training protocol. The speed of the treadmill was gradually increased until the animals were running at the designated speed. The training program consisted of running at 30 m/min for 30 min/day at 0% grade level, 5 days per week, once a day for 6 weeks in the exercising groups. Running test was conducted between the hours 13:00–16:00 (to ignore basal glucocorticoid activity). Rats of the control group were just kept sedentary on the treadmill.

### Sample collection and biochemical analysis

At the end of the experiment, after 12-h starvation, blood, liver and muscle samples were collected by decapitation through cervical dislocation. Blood samples were centrifuged at 5000 rpm at 4 °C for 10 min in a refrigerated centrifuge (Universal 320R, Hettich, Germany) with biochemical gel tubes (Standardplus & Medical Co., Ltd., Germany) to obtain serum samples. In addition, the tissue samples were preserved at − 80 °C in the deep freeze (Hettich, Germany) until their analysis. Glucose (GLU), total cholesterol, High-Density Lipoprotein cholesterol (HDL) and triglycerides (TG) levels were measured with an automatic biochemical analyzer (Samsung Labgeo PT10). Aspartate aminotransferase (AST) and alanine aminotransferase (ALT) levels were also measured to evaluate any toxic effect of CrHis and biotin on liver cells. For determination of Cr concentrations, 0.3 g feed samples were first digested with 5 mL concentrated HNO3 in a Microwave Digestion System (Berghoff, Eningen, Germany) for 30 min. The specimens were subjected to graphite furnace atomic absorption spectrophotometer (AAS, Perkin-Elmer, Analyst 800, Norwalk, CT). The measurement of biotin in the diet was detected with a coupled HPLC/competitive binding assay as previously described with minor modifications [40]. The reversed-phase column used was a Sphereclone 250 × 4.6 mm, and the biotin-containing chromatography fractions were dried under a stream of nitrogen before the assay.

### RNA extraction and quantitative real-time polymerase chain reaction

Total RNAs were isolated from the liver and muscle tissues by the RNeasy total RNA isolation kit (QIAGEN, Hilden, Germany) as defined according to the manufacturer protocol. After isolation, the concentration of total RNA was measured on a Nanodrop spectrophotometer (Maestrogen Inc., Taiwan). Then, 1 *μ*g of total RNA was reverse-transcribed to cDNA using commercial first-strand cDNA synthesis kit (QIAGEN, Hilden, Germany). Expression IRS-1, PPAR훾, NF휅B genes were detected with real-time polymerase chain reaction. For this purpose, 5 *μ*L cDNA, 12.5 *μ*L 2X SYBR Green Master Mix (QIAGEN Fast Start Universal SYBR Green Master Mix), and primer pairs at 0.5 *μ*M concentrations in a final volume of 10*μ*L were mixed and qRT-PCR (LightCycler480 II, QIAGEN, Hilden, Germany) was performed as follows: initial denaturation at 95^∘^C for 10 min, denaturation at 95^∘^C for 15 s, annealing at 65^∘^C for 30 s, and extension at 72^∘^C for 15 s with 40 repeated thermal cycles measuring the green fluorescence at the end of each extension step [41]. Glyceraldehyde 3-phosphate dehydrogenase (GAPDH) was also amplified from the samples and served as the housekeeping gene. The primer sequences are listed in Table 1. The relative expression of genes with respect to GAPDH was calculated with the efficiency corrected advance relative quantification tool provided by the software (https://www.qiagen.com/us/geneglobe/ Data Analysis Center). Gene expression profile was assessed as ΔCT values. Gene expression fold changes were presented as relative to the control and calculated using 2 ^−ΔΔCT^ method. The differential gene expression was rated in pairs with fold-change cut-off of 2 and significance value of *P* < 0.05.

**Table 1:** Primer sequences of genes used for real-time polymerase chain reaction

Gene	Primer	Sequence
IRS-1	Forward	5′-GCGCAGGCACCATCTCAACAACC-3′
	Reverse	5′-GCACGCACCCGGAAGGAACC-3′
PPAR-γ	Forward	5′-TCAAACCCTTTACCACGGTT-3′
	Reverse	5′-CAGGCTCTACTTTGATCGCA-3′
NF-κB	Forward	5′-TGAGGCTGTTTGGTT TGAGA −3′
	Reverse	5′-TTATGGCTGAGGTCTGGTCTG-3′
GAPDH	Forward	5′-TGATGACATCAAGAAGGTGGTGAAG-3′
	Reverse	5′TCCTTGGAGGCCATGTGGGCCAT-3′

*IRS-1* insulin receptor substrate-1, *PPAR-γ* peroxisome proliferator-activated receptor gamma,

*NF-κB* nuclear factor kappa B

### Statistical analysis

The sample size was calculated based on a power of 85% and an error of 0.05. The data were evaluated using the ANOVA procedure in the IBM SPSS (version 22) package program. Comparisons between groups were done by the Tukey Post Hoc test. For associations among variables, the Pearson Correlation Test was performed. Cohen’s D effect size calculations were also done to determine the effect of supplementation on glucose and triglyceride levels and protein expressions (effect size ≤0.2, small; 0.2, effect size ≤0.6, moderate; effect size ≤0.6, large). The data were given as group mean and standard error of the mean (SEM). Differences were considered significant at *p*-values < 0.05.

## Results

### Effects on body weight and Cr and biotin intake

The body weight of the rats differs between the groups (Table 2). Final body weight decreased in exercise and combination of regular exercise and CrHis and CrHis+Bio groups compared to the sedentary control group (*p* < 0.01).The final body weight was within the range of 270.86 to 312.29 g. The daily Cr and biotin intake were significantly higher in rats fed Cr and biotin supplemented diets with exercise training than in other groups (*p* < 0.01).

**Table 2:** Effects of chromium histidinate and biotin on body weight, Cr and biotin intake and serum parameters in exercise-trained rats

Parameters	C	CrHis	Bio	CrHis+Bio	E	E + CrHis	E + Bio	E + Bio + CrHis
Final body weight, g	312.29 ± 4.33^a^	301.86 ± 2.47^a^	307.00 ± 4.20^a^	301.43 ± 6.01^a^	282.29 ± 2.62^b^	270.86 ± 2.48^b^	276.71 ± 4.93^b^	273.29 ± 3.23^b^
Cr intake, μg/rat/day	23.52 ± 1.15^d^	32.45 ± 2.07^b^	24.64 ± 1.83^c^	32.43 ± 2.30^b^	25.76 ± 1.46^c^	35.4 ± 2.33^a^	26.88 ± 2.91^c^	36.88 ± 3.15^a^
Biotin intake, μg/rat/day	5.46 ± 0.82^c^	5.72 ± 0.75^c^	135.3 ± 4.26^b^	135.1 ± 3.85^b^	5.98 ± 0.68^c^	6.24 ± 1.33^c^	147.5 ± 6.55^a^	153.75 ± 8.70^a^
Glucose, mg/dL	121.50 ± 3.53^a^	110.71 ± 1.67^b^	118.67 ± 1.48^b^	106.86 ± 1.03^c^	112.29 ± 2.67^b^	97.71 ± 1.95^d^	109.29 ± 1.51^b^	93.57 ± 1.25^e^
Cholesterol, mg/dl	86.72 ± 3.23^a^	78.25 ± 2.61^b^	80.55 ± 3.42^b^	75.15 ± 3.20^c^	77.43 ± 1.95^b^	71.86 ± 2.35^d^	76.99 ± 2.83^b^	67.55 ± 2.85^e^
HDL, mg/dL	11.48 ± 0.44^d^	14.29 ± 0.42^c^	13.32 ± 0.42^cd^	15.86 ± 0.63^c^	15.29 ± 0.78^c^	18.14 ± 0.55^b^	16.14 ± 0.59^c^	22.71 ± 1.43^a^
Triglyceride, mg/dL	139.00 ± 9.84^a^	91.57 ± 4.85^c^	108.67 ± 5.74^b^	77.00 ± 5.23^d^	68.43 ± 3.05^e^	61.14 ± 2.82^ef^	65.71 ± 6.28^e^	51.71 ± 4.45^g^
AST, U/L	245.67 ± 44.12	240.71 ± 16.38	239.00 ± 20.12	237.29 ± 17.02	239.71 ± 15.48	243.71 ± 19.67	242.43 ± 11.29	236.43 ± 21.01
ALT, U/L	102.00 ± 3.39	100.71 ± 4.32	103.17 ± 7.12	104.57 ± 4.45	106.29 ± 5.36	103.14 ± 2.76	105.29 ± 4.55	103.29 ± 3.90

Data are given as mean ± standard error. (a-g) The difference between the groups with different letters is statistically significant (*p* < 0.05). *C* Control, *CrHis* Chromium histidinate, *Bio* Biotin, *E* Exercise, *HDL* High-Density Lipoprotein, *AST* Aspartate aminotransferase, *ALT* Alanine aminotransferase

### Effects on blood glucose level

As shown in Table 2, the blood glucose level was found to be significantly (*p* < 0 .001) lowered in CrHis and CrHis+Bio groups compared to the sedentary control group. No significant change in blood glucose values was observed between biotin and control groups. Regarding the exercise-trained rats, we observed that E + CrHis and E + Bio + CrHis groups exhibited a significant decrease in blood glucose level compared with the exercise group (*p* < 0 .001). Exercise-trained rats supplemented with biotin had no significant change in blood glucose level. Blood glucose level was decreased by 10.69% and 16.67% in Bio + CrHis and E + Bio + CrHis groups respectively, compared to their respective control groups (effect size = large; ƞ2 = 0.773). Interestingly, the combination of CrHis and biotin was more efficient in reducing blood glucose level in sedentary and exercised rats, compared to CrHis alone. However, CrHis and biotin exhibited the highest effects in exercised rats, compared to the sedentary rats (Table 2).

### Effects on cholesterol and triglycerides levels

Total cholesterol and triglycerides levels were found to be significantly (*p* < 0.05) lowered in CrHis and CrHis+Bio groups. In no exercised rat, High-Density Lipoprotein Cholesterol (HDL) was increased in CrHis and CrHis+Bio groups compared to the control group. No significant change in HDL cholesterol and triglycerides concentrations were observed in rats treated with biotin alone, compared with the control group. Exercise significantly (*p* < 0.001) increased HDL cholesterol, but decreased cholesterol and triglycerides levels compared with the sedentary control group. We also observed that HDL cholesterol level significantly (*p* < 0.001) increased in E + CrHis and E + CrHis+Bio groups while cholesterol and triglycerides levels decreased in the same groups compared with the exercise group. Triglycerides levels were decreased in sedentary (44.60%) and exercised (24.43%) rats supplemented with both CrHis and biotin (effect size = large; ƞ2 = 0.802) while HDL cholesterol was elevated in both groups (27.61% in CrHis+Bio and 32.67% in E + CrHis+Bio) (effect size = large; ƞ2 = 0.756). In sedentary and exercised rats, the combination of CrHis and biotin exhibited the highest effects, compared with CrHis alone. The lowest value of total cholesterol and triglycerides levels were found in the CrHis+Bio group, but the highest HDL cholesterol was observed in the E + CrHis+Bio group (Table 2).

### Effects on aspartate aminotransferase and alanine aminotransferase levels

Throughout the study, aspartate aminotransferase (AST) and alanine aminotransferase (ALT) levels were statistically unchanged in all groups after treatment (Table 2; effect size: none; ƞ2 = 0.003 and ƞ2 = 0.032).

### Effects on IRS-1, PPAR-γ and NF-κB expressions

**Fig. 1:**
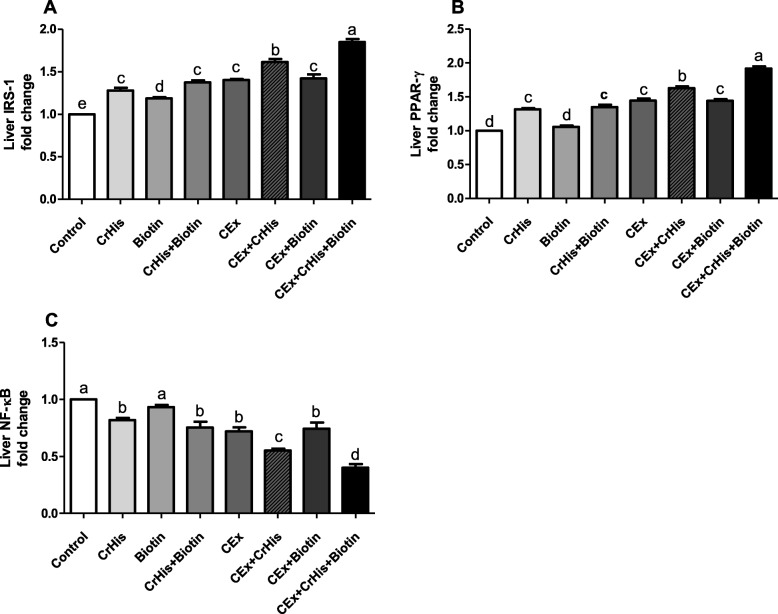
Effects of chromium histidinate and biotin supplementation on the expressions of IRS-1 (Panel **a**), PPAR-γ (Panel **b**) and NF-κB (Panel **c**) in the liver of sedentary and exercise-trained rats. Different superscripts (a–e) indicate group mean differences (*p* < 0.05)

**Fig. 2:**
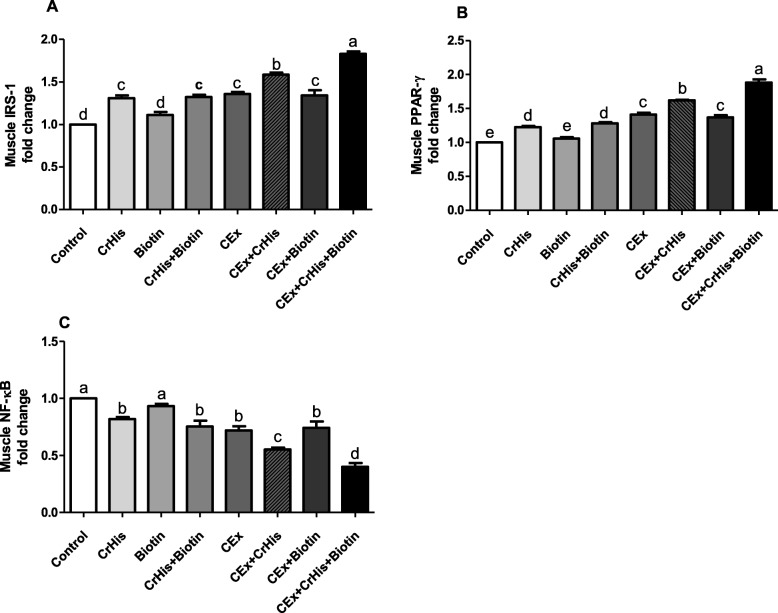
Effects of chromium histidinate and biotin supplementation on the expressions of IRS-1 (Panel **a**), PPAR-γ (Panel **b**) and NF-κB (Panel **c**) in the muscle of sedentary and exercise-trained rats. Different superscripts (a–e) indicate group mean differences (*p* < 0.05)

The effects of CrHis and biotin supplementation on the expression of IRS-1, PPAR-γ and NF-κB proteins in the liver and muscle in sedentary and exercise-trained rats are shown in Figs. 1 and 2. When compared with the sedentary control group, the protein expression levels of IRS-1 in the liver and muscle were significantly increased in the CrHis and CrHis+Bio groups (effect size: large; ƞ2:0.973 for liver and ƞ2 = 0.965 for muscle). In exercise-trained rats treated with biotin (E + Bio), there was no significant difference in IRS-1 expression. In contrary, E + CrHis and E + CrHis+Bio groups presented an increased level of IRS-1 compared with the exercise group. Moreover, both CrHis and biotin were more efficient in increasing the IRS-1 level in exercised rats than in sedentary rats. (Fig. 1a and Fig. 2a).

Only sedentary and exercise-trained rats treated with CrHis and CrHis+biotin exhibited a significant increase in PPAR-γ expression level in the liver and muscle compared with the sedentary control group or exercise group **(**effect size: large; ƞ2 = 0.986 for liver and ƞ2 = 0.983 for muscle). The most important change in PPAR-γ expression level was found in E + CrHis+Bio groups (*p* < 0.05) (Fig. 1b and Fig. 2b).

Regarding the expression of NF-κB protein in the liver and muscle of no exercised rats, we found that its level was significantly decreased in CrHis and CrHis+Bio groups compared with the control group. Similarly, in exercise-trained rats, CrHis alone and CrHis+Bio significantly decreased the expression of NF-κB in the liver and muscle, with the highest effect when used together **(**effect size: large; ƞ2 = 0.953 for liver ƞ2 = 0.950 for muscle). No significant difference in liver and muscle NF-κB expression level was found in sedentary and exercised rats supplemented with biotin alone, compared to the sedentary group and exercised group, respectively. The efficacy of CrHis and biotin, as well as their combination, were more remarkable in exercised rats than in sedentary rats (Fig. 1c and Fig. 2c).

Correlations among response variables were statistically significant (Table 3). There were negative correlations between glucose concentration with serum HDL concentration (*r* = − 0,746), and expressions of liver and muscle IRS (*r* = − 0.852 and *r* = − 0.854), as well as liver and muscle PPAR-γ (*r* = − 0.878 and *r* = − 0.869). In the contrary, we found a positive correlation between glucose concentration and triglyceride concentration (*r* = 0,732), and expressions of liver and muscle NF-κB (*r* = 0.904 and r = 0.904). Serum HDL cholesterol was negatively correlated with serum triglyceride (r = − 0,626), AST (r = − 0,138), ALT (r = − 0,049) and liver and muscle NF-κB (r = − 0,881and r = − 0,881), but positively correlated with liver and muscle IRS (r = 0,855 and r = 0,809) and PPAR-γ (r = 0,849 and r = 0,817). Serum triglyceride concentration was correlated with expression of liver and muscle IRS (*r* = − 0.797 and *r* = − 0.790), PPAR-γ (*r* = − 0.782 and *r* = − 0.774) and NF-κB (*r* = 0.750 and *r* = − 0.750). Moreover, expression of liver and muscle IRS and PPARg were negatively correlated with expressions of NF-κB, whereas IRS was positively correlated with expressions of PPAR-γ.

**Table 3:** Pearson’s correlation coefficients (r) among variable

	HDL	Triglyceride	AST	ALT	Liver IRS	Liver PPARγ	Liver NF-κB	Muscle IRS	Muscle PPARγ	Muscle NF-κB
Glucose	− 0,746**	0,732**	0,045	0,018	−0,852**	−0,878**	0,904**	−0,854**	− 0,869**	0,904**
HDL		−0,626**	−0,138	− 0,049	0,855**	0,849**	−0,881**	0,809**	0,817**	−0,881**
Triglyceride			−0,088	−0,095	− 0,797**	−0,782**	0,750**	−0,790**	− 0,774**	0,750**
AST				0,176	0,290	0,253	−0,280	0,321	0,270	−0,280
ALT					0,265	0,246	−0,242	0,195	0,266	−0,242
Liver IRS						0,955**	−0,953**	0,970**	0,960**	−0,953**
Liver PPARγ							−0,955**	0,965**	0,984**	−0,955**
Liver NF-κB								−0,946**	− 0,956**	0,999**
Muscle IRS									0,972**	−0,946**
Muscle PPARγ										−0,956**

*HDL:* High-Density Lipoprotein, *AST:* Aspartate aminotransferase, *ALT:* Alanine aminotransferase. PPAR-γ: peroxisome proliferator-activated receptor gamma, IRS-1: insulin receptor substrate-1, NF-κB: nuclear transcription factor kappa B

** Correlation is significant at the 0.01 level.

## Discussion

To the best of our knowledge, this is the first study to evaluate the effects of dietary chromium histidinate (CrHis) and biotin supplementation on liver transaminases, serum glucose and lipid levels, and proteins expression levels of IRS-1, PPAR-γ, and NF-κB in liver and muscle of exercised rats. We observed that CrHis/biotin supplementation significantly decrease glucose, cholesterol, triglyceride, and NF-κB levels, but increase serum HDL cholesterol, IRS-1 and PPAR-γ expression in the liver and muscle of exercise-trained rats. Interestingly, the beneficial effects of CrHis and biotin on all metabolic parameters were more pronounced when they were administered together.

Exercise is considered a very important tool in the prevention and treatment of various diseases [3, 4, 42]. Numerous studies show that exercise decreases blood glucose level, improves insulin sensitivity, increases the rate of fat oxidation and ameliorates postprandial triglyceride response [1, 43, 44]. However, in the current study, exercised animals exhibited a significant decrease in glucose, total cholesterol and triglyceride levels, but an increase in HDL cholesterol compared to the sedentary control group. Leon et al. [16] have shown that a 12-week exercise decreased cholesterol and TG concentrations and increased HDL cholesterol levels in rats. In another similar work, Leon and Sanchez [45] reported that exercise-induced a decrease in LDL and TG levels, but had no effect on blood total cholesterol concentration. The beneficial effect of exercise on the risk of metabolic diseases may be due to the improvement in glucose and insulin sensitivities, inflammatory markers and blood lipids level [46, 47]. Exercise combined with micronutrient supplementation such as CrHis and biotin are efficient in preventing or treating various metabolic diseases [48, 49]. Chromium (Cr) is an essential trace element particularly involved in carbohydrate, fat, and protein metabolism [27, 50]. Previous works have shown that chromium picolinate (CrPic) supplementation modulated insulin, glucose and lipid metabolism in type 2 diabetic rats [31]. Moreover, Grant et al. [51] reported that exercise training combined with Cr supplementation is more beneficial than exercise training alone in improving various metabolic parameters. Biotin, a water-soluble vitamin, is an integral component of carboxylation reactions involved in glucose and insulin metabolism. Fernandez-Mejia [32] and Osada et al. [52] have reported that biotin deficiency induced fatigue in mice. In addition, Cr and biotin supplementation have been shown to modulate various metabolic pathways such as insulin signalling pathway in patients who are deficient [31, 32]. It was reported that CrPic and biotin supplementation improves glycemic control [25, 34, 53] and modulates lipid pathways [33] in people with type 2 diabetes. Also, some clinical studies indicated that a combination of CrPic and biotin was more efficient in modulating glucose and lipid metabolism in diabetic patients [25, 53, 54]. Similarly, we reported in our previous studies that the combination of CrPic and biotin was efficient in lowering the blood glucose level in heat-stressed quail [55–57]. In the present study, we found that CrHis and biotin supplementation led to a significant decrease in glucose and triglyceride levels, but an increase in HDL cholesterol compared with the exercise group. The lowest concentrations of triglycerides and glucose, as well as the highest level of HDL cholesterol, were found in exercise-trained rats treated with both CrHis and biotin. In parallel, previous studies reported that the combination of Cr and biotin is more efficient in modulating insulin, glucose and lipid metabolism in type 2 diabetes patients [25, 53, 54]. Because transaminases levels in the liver are considered a key factor when evaluating the cytotoxicity of a drug, the non- significant change in AST and ALT levels in all groups indicates no harmful effects on hepatocyte cells.

Peroxisome proliferator-activated receptor gamma (PPAR-γ), insulin receptor substrate-1 (IRS-1) and nuclear transcription factor kappa B (NF-κB) are important biomarkers involved in numerous metabolic processes. PPAR-γ plays a key role in regulating lipid, carbohydrate, glucose and insulin metabolisms [58]. It has been shown that exercise induced an increase in PPAR-γ expression in liver [59, 60] and skeletal muscle tissues [61]. In the current study, PPAR-γ expression levels in the liver and muscle tissues were significantly elevated compared to the control group. Remarkably, CrHis and biotin supplementation significantly increased PPAR-γ expression levels in sedentary and exercised rats. The efficacy of CrHis and biotin was more pronounced when used simultaneously, thus indicating a synergetic effect. Our previous findings demonstrated that CrPic and biotin, as well as their combination, increased PPAR-γ expression in adipose tissue and improved insulin resistance in type 2 diabetes rats [62].

Insulin receptor substrate-1 (IRS-1) is involved in metabolic and mitogenic effects of insulin [63, 64]. Numerous studies have reported that exercise increased IRS-1 expression in human skeletal muscle [22, 65]. Similarly, we observed in the present study that exercise rats exhibited an increase in IRS-1 expression in the muscle and liver tissues compared to the sedentary control group. Moreover, in sedentary and exercised rats, CrHis and biotin treatment induced a significant increase in IRS-1 expression in the muscle and liver tissues, compared to their respective control groups. These findings may suggest that the beneficial effect of CrHis and biotin on lipid and glucose metabolism was probably associated with the improvement of insulin signal transduction in target tissues. These results corroborate previous works published by Jain et al. [66] who reported that IRS-1 expression in the liver tissues of type 2 diabetic rats increased after treatment with chromium dinicocysteinate. In other similar studies, CrPic supplementation improved glucose disposal rates and significant increased IRS-1 expression and phosphatidylinositol-3 kinase activity in skeletal muscles in obese rats [35].

Nuclear transcription factor kappa B (NF-κB) is a transcriptional factor particularly involved in the inflammatory process. In the current study, the decrease in NF-κB expression in untreated exercised rats is similar to the previous study, who reported that treadmill training reduced the overexpression of NF-κB in rat brain tissue [67]. Moreover, CrHis and biotin, as well as their combination, induced a significant decrease in NF-κB level in the muscle and liver tissues, compared with the exercise or control group. However, the capacity of these micronutrients in lowering NF-κB expression was more pronounced when CrHis and biotin were administered together. In parallel with the results of the current study, we recently reported a greater reduction in NF-κB expression in diabetic rats supplemented with CrHis [68]. On the contrary, Kuhad et al. [69] reported that NF-κB subunit was significantly elevated in the kidneys of diabetic rats after treatment with CrPic. Because NF-κB level is an indicator of the inflammatory response [70], decreased NF-κB expression in the muscles of rats supplemented with CrHis and biotin may indicate an anti-inflammatory property.

## Conclusion

The present study demonstrates that dietary CrHis/biotin supplementation improved glucose metabolism and lipid profile as well as the proteins expression levels of IRS-1, PPAR-γ, and NF-κB in the muscle and liver of sedentary and exercise-trained rats. The efficacy of CrHis/biotin was more efficient in exercised rats. Further studies on the effects of these nutrients on metabolic risk factors are highly needed.

## Abbreviations

ALT: Alanine aminotransferase
AST: Aspartate aminotransferase
CrHis: Chromium histidinate
CrPic: Chromium picolinate
GLU: Glucose
HDL: High-density lipoprotein
IRS-1: Insulin receptor substrate-1
NF-κB: Nuclear factor kappa B
PPAR-γ: Peroxisome proliferator-activated receptor gamma
TG: Triglyceride
